# When helping helps: exploring health benefits of cancer survivors participating in for-cause physical activity events

**DOI:** 10.1186/s12889-018-5559-6

**Published:** 2018-05-29

**Authors:** M. Renée Umstattd Meyer, Andrew R. Meyer, Cindy Wu, John Bernhart

**Affiliations:** 1 Department of Health, Human Performance, & Recreation, Robbins College of Health and Human Sciences, Baylor University, One Bear Place #970311, Waco, TX 76798 USA; 2 Department of Management, Hankamer School of Business, Baylor University, One Bear Place #98006, Waco, TX 76798 USA; 3 Department of Exercise Science, Arnold School of Public Health, University of South Carolina, 921 Assembly Street, PHRC, 1st Floor, Columbia, SC 29208 USA

**Keywords:** Physical philanthropy, Muscular Christianity, Non-profit organizations, Volunteerism, Pro-social behaviors, Exercise

## Abstract

**Background:**

Over 15.5 million Americans live with cancer and 5-year survival rates have risen to 69%. Evidence supports important health benefits of regular physical activity for cancer survivors, including increased strength and quality of life, and reduced fatigue, recurrence, and mortality. However, physical activity participation among cancer survivors remains low. Cancer organizations provide various resources and support for cancer survivors, including emotional, instrumental, informational, and appraisal support. Many cancer organizations, like the LIVE**STRONG** Foundation, support the cancer community by sponsoring and hosting for-cause physical activity events, providing opportunities for anyone (including cancer survivors) to “help”/support those living with cancer. The concept of *helping others* has been positively related with wellbeing, physical activity, and multiple health behaviors for those helping. However, the role of helping others has not been examined in the context of being physically active to help others or its relationship with overall physical activity and quality of life among those helping. Therefore, we developed a path model to examine relationships between cancer survivors’ (1) desire to help others with cancer, (2) physically active LIVE**STRONG** participation to help others, (3) regular physical activity engagement, and (4) quality of life.

**Methods:**

In 2010, 3257 cancer survivors responded to an online survey sent to all people involved with the LIVE**STRONG** organization at any level. The hypothesized path model was tested using path analysis (Mplus 8).

**Results:**

After list-wise deletion of missing responses, our final sample size was 3122 (61.8% female, mean age: 48.2 years [SD = 12.7]). Results indicated that the model yielded perfect fit indexes. Controlling for age, sex, income, and survivorship length, desire to help was positively related with physically active LIVE**STRONG** participation (β = .11, *p* < .001), which was positively related with regular physical activity (β = .30, *p* < .001), and regular physical activity was positively related with quality of life (β = .194, *p* < .001).

**Conclusions:**

Results suggest that cancer survivors can benefit from participating in for-cause physical activity events, including more regular physical activity. Researchers need to further investigate the role of helping others when examining health behaviors and outcomes, and cancer organizations should continue encouraging cancer survivors to help others by participating in physical activity events.

## Background

Over 15.5 million American children and adults were living with a history of cancer on January 1, 2016, and 5-year survival rates have risen to 69% for all cancers diagnosed between 2005 and 2011 [1]. Evidence supports important health benefits of regular physical activity for cancer survivors, including increased strength, quality of life, and self-esteem; fewer negative treatment-related side-effects; and reduced fatigue, anxiety, recurrence, and mortality [2–5]. Additionally, poorer health indicators (e.g., poorer physical functioning, physical health, and general health), greater fatigue, and lower health-related quality of life are associated with increased sedentary behaviors seen among cancer survivors, independent of physical activity participation [5–7]. In fact, physical inactivity among cancer survivors has been suggested to contribute to increased risk of second primary malignancies [8]. However, physical activity participation among cancer survivors remains low [7, 9, 10], with evidence indicating that up to 88% of cancer survivors are inactive. Additionally, cancer survivors report spending the majority of their waking time in sedentary behaviors [7, 11].

Cancer organizations, like the LIVE**STRONG** Foundation, provide various kinds of resources and support for cancer survivors, including emotional, instrumental, informational, and appraisal support in the forms of online communities, in-person events, and educational information on topics ranging from prevention to treatment. Another way cancer organizations support the cancer community is through hosting for-cause physical activity events, providing opportunities for anyone (including cancer survivors) to engage in physical activity and “help”/support those living with cancer while building community and fundraising for the cause. Examples of for-cause physical activity events include 5 k–10 k runs or walks, marathons, triathlons, and cycling rides to raise awareness of and/or financial support for the identified cause. Recent research examining relationships between participation in large physical activity events (e.g., cycling events, triathlons, 5 k, 8 k, half marathons, marathons, etc..), which could include for-cause physical activity events, demonstrates an increase in physical activity for many participants during training months leading up to the event, and maintenance of or increase in physical activity after an event concludes [12, 13]. The combination of promoting notions of helping others while being physically active provides a unique context in which to encourage multiple health-enhancing behaviors: physical activity and helping others.

The concept of *helping others* has been examined in the fields of volunteerism, spirituality, and work in the broader umbrella-area of pro-social behavior (intentional actions undertaken to help or benefit others) [14, 15]. Collectively, helping others has been associated with multiple health-enhancing behaviors, positive wellbeing, social support, confidence, enriched life purpose, and physical, mental, and emotional health-related benefits for individuals across age ranges [14–26]. Additionally, previous work also supports mental, emotional, and social health related benefits of helping others for those living with a disease or chronic physical condition [27–29].

To summarize, helping others seems to benefit the wellbeing of helpers, including helpers with cancer and other illnesses, although mechanisms that facilitate these benefits are not often examined or well understood. Additionally, training for and participating in for-cause physical activity events is related with participants engaging in regular physical activity during training and continuing afterwards, promoting regular physical activity participation [12, 13]. One mechanism with the potential to positively impact regular physical activity and associated benefits, including quality of life, is the idea that people, including cancer survivors, want to help others through physical activity by using their physical bodies [30]. While this has not been empirically examined, to some degree it is practically supported when looking at the number of for-cause physical activity events sponsored by for-cause organizations [30] and theoretically supported through the historical lens of muscular Christianity, which heavily influenced modern sport and physical activity ideals, and includes a core tenant of “doing good for others” (to help others) using our physical “God-given” bodies to do so [31, 32]. While this historical socio-theological movement and its associated ideologies is often cursorily discussed in modern sport contexts [33], sports historians, sport sociologists, and religious sport scholars highlight the influence muscular Christianity had in shaping sporting ideals, and posit that these can be observed more broadly in social ideals, health, physical activity, and contemporary physical activity in education [32, 34–39].

The core ideals of muscular Christianity can be found in the writings of British Victorian authors and clergymen, namely Charles Kingsley and Thomas Hughes. In his popular novel *Tom Brown at Oxford* (1861) Hughes states that muscular Christians are “to have strong and well-exercised bodies…that a man’s body is given to him to be trained and brought into subjection, and then used for the protection of the weak, the advancement of all righteous causes, and the subduing of the earth which God has given to the children of men” (p. 82) [31]. While the term muscular Christianity is relegated to historical discussions of sport and physical activity, the tenants of the historical movement are found in sport and physical activity ideals today [40].

Related to the focus of this current study, existing research parallel’s tenants of muscular Christianity, demonstrating its relevance today in various sport and health related areas. For example, Hoverd and Sibley (2007) identified how “conceptions of health use moral discourses derived from Christianity” (p. 391) [41], and recent empirical studies have focused on identifying muscular Christian themes among various populations with relation to place attachment and prescription drug misuse [40]. Additional research has discussed the ways in which moral conversations about bodies have been shaped by Christian ideals since the nineteenth century [41]. For example, Townsend (2009) describes “the moralized understanding of obesity,” and explores how media perpetuates these moralistic ideas associated with obesity, often ignoring the “structures which create ill health” (p. 172) [42]. Gerber (2009) also addresses moral components of health and physical activity, identifying how formatively religion influenced norms of weight loss and contributed to “moral discourses on fat” (p. 406) [43].

Research has also identified Christian moral ideals among community soup kitchen volunteers, an example of attaching religious aspects of helping others in secular contexts [44]. Additionally, Christian-founded values and ideals have been examined and continue to perpetuate our culture, with recent work identifying media representations of Christian football and soccer players in the United States and Germany [45]. Christian themes have also recently been explored within CrossFit settings where participants describe gym spaces and compare them to church [46]. Critics of such moral language surrounding body, health, and physical activity, such as Hoverd and Sibley (2007) state, “the haphazard application of Christian moral discourse in everyday contexts to evaluate the body and health behaviors is generalized, nontheologically coherent and…reflects a morally loaded discourse in health” (p. 391) [41]. This current paper acknowledges the history and contemporary influence of religious ideals in constructing meaning for participants at LIVE**STRONG** for-cause physical activity events. While other motives for participation in for-cause physically activity events by cancer survivors may exist, this research focused on the idea of helping others, derived from the nineteenth century muscular Christian movement, and seen in other fields.

To further examine and describe the potential role of using our physical bodies as a means to “help others” and as a mechanism to improve overall physical activity engagement and general wellbeing for people with cancer, we developed a path model to examine relationships between cancer survivors’ (1) desire to help others with cancer, (2) physical activity participation in LIVE**STRONG** to help others with cancer, (3) regular physical activity engagement, and (4) quality of life.

## Methods

### Data collection

Although previously reported [47], a description of survey design and administration will be briefly summarized here. People involved with the LIVE**STRONG** organization at any level during 2010 were invited to participate in an anonymous online survey by the (then named) Lance Armstrong Foundation. Estimated survey completion time was between 5 and 30 min based on the respondent’s connection with cancer. Recruitment occurred through email, Twitter, and Facebook, and in collaboration with several comprehensive cancer centers who shared the survey with patients. The online survey was available on the LIVE**STRONG**.org website and was reviewed and approved by the Western Institutional Review Board. Informed consent to participate in the study was obtained using the following statement on the first page of the online survey **“**By answering the survey questions you acknowledge your consent to participate in this study.” Survey participation occurred between June 2010 and April 2011 and included respondents who had been personally diagnosed with cancer, as well as those who had never been diagnosed with cancer. Only respondents who had been diagnosed with cancer in their lifetime, and who were not currently receiving hospice or palliative care, are included in the current analyses (*n* = 3257).

### Measures

The following socio-demographic items from the 2010 LIVE**STRONG** survey were used to describe the sample: gender, age, race/ethnicity, marital status, education, income, U.S. residency, and survivorship length. Desire to help others was measured using a single item asking if a respondent would like to do more to help other people affected by cancer. As part of the survey, respondents were also asked to indicate their type of involvement with LIVE**STRONG**, which included a variety of options (e.g., volunteering at LIVE**STRONG** events, making a financial donation, being a fan of LIVE**STRONG** on Facebook, being physically active in a LIVE**STRONG** cause-related event, etc.…). “Physically active participation” in LIVE**STRONG** was defined as participation in at least one of the following LIVE**STRONG** events that required the person to engage in physical activity and/or exercise (e.g., running, cycling, and/or swimming): LIVE**STRONG** Challenge, Ride for the Roses, the Register’s Annual Great Bicycle Ride Across Iowa (RAGBRAI) a non-competitive cross-state bicycle ride organized by *The Des Moines Register*, running in a LIVE**STRONG** sponsored marathon, or an item labeled “participated in other Team LIVE**STRONG** events (i.e., triathlon, running relay…).” Overall physical activity engagement was measured using a single item asking respondents to indicate if the following statement was true since completing treatment for cancer, “I have participated in regular physical activity (For example, you participate in some type of physical activity at least 2 to 3 times a week)”. Quality of life was measured using 31 items from the original Quality of Life in Adult Cancer Survivors (QLACS) scale [48], which were used to calculate an overall quality of life summary score for each respondent and included questions from all five of the originally developed cancer-specific domains (appearance concerns, financial problems, distress over recurrence, family-related distress, and benefits of cancer) and six generic quality of life domains (negative feelings, cognitive problems, sexual problems, physical pain, fatigue, and social avoidance). Original QLACS scale development indicated good internal consistency and validity through factor analysis with cancer survivors [48]. However, unlike the original scale, QLACS items had dichotomous response options (yes/no) in the LIVE**STRONG** survey. The range of scores for the QLACS summary variable in this study was 0–31, with 31 indicating greater quality of life.

### Data analysis

**Fig. 1:**
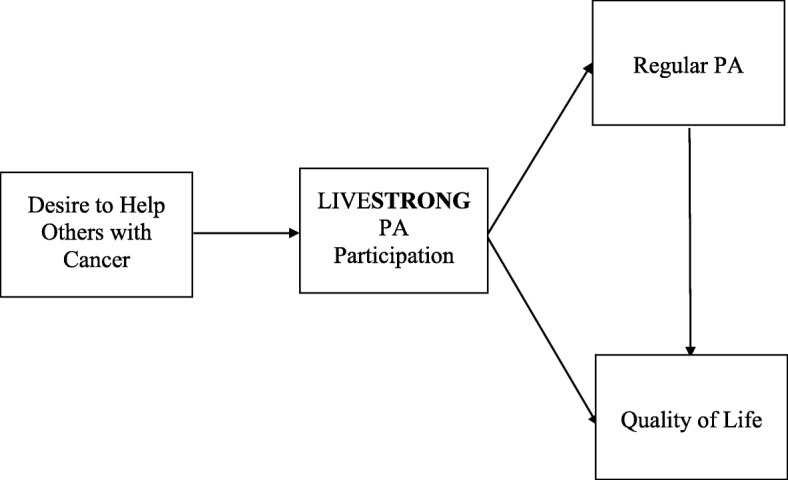
Summary of the proposed relationships in the path model. PA = physical activity

To examine the proposed relationships among the variables of interest, we conducted a path analysis with a robust weighted least square estimator using Mplus 8 [49]. The path model allows us to control for and model the correlations among these variables simultaneously. The robust weighted least square estimator treats the binary endogenous variables (i.e., physical activity participation in LIVE**STRONG** and regular physical activity engagement) as continuous latent response variables and thus approaches asymptotic normality. Specifically, our path model was developed to examine (1) whether cancer survivors’ desire to help others with cancer was related to their physically active participation in LIVE**STRONG**, (2) whether cancer survivors’ physically active participation in LIVE**STRONG** to help others with cancer was related to their regular physical activity engagement, (3) whether cancer survivors’ physically active participation in LIVE**STRONG** was related to their quality of life, and (4) whether cancer survivors’ regular physical activity engagement was related to their quality of life. Furthermore, in-line with the basic tenant of muscular Christianity’s argument, we tested the mediating roles of physically active participation in LIVE**STRONG** events and regular physical activity engagement by examining the indirect effect of cancer survivors’ desire to help others on their quality of life (see Loeys, Moerkerke, and Vansteelandt, 2015 [50]). To counter the non-normal distribution issue in testing the indirect effect, we examined bias-corrected bootstrapping confidence intervals [51] to determine if the indirect effect estimate was significantly different from zero. In addition, following Imai, Keele, and Tingley’s (2010) suggestion, we conducted a sensitivity analysis on the indirect effect to determine its robustness against the unobserved confound that may have inflated the reported relationship between the mediators and outcome variable [52]. Finally, because previous research has reported that age, sex, income, and length of cancer survivorship were related with level of physical activity and quality of life [53–60], we controlled for this socio-demographic information in our analysis to account for its potential influence (for clarity of the reporting, the control variables were omitted in the figures). The hypothesized model is depicted in Fig. 1.

## Results

### Sample description

LIVE**STRONG** survey respondents in 2010 included 12,037 people, of which 98.4% fully completed the online survey (1.6% completed between 83 and 99% of the survey). Of these respondents 27.5% (*n* = 3257) identified their current stage of survivorship as “having finished treatment”. Of these cancer survivors 62.1% were female and middle age (mean age = 48.2, SD = 12.7). Only 11.4% (*n* = 370) of survivors reported actively participating in any LIVE**STRONG** physical activity events (e.g., LIVE**STRONG** Challenge, triathlon…). Because the total number of cases containing missing responses in our study variables (including control variables) was relatively small (*n* = 135, 4% of the original sample size of 3257), we considered the missing response pattern inconsequential [61] and therefore deleted these cases list-wise. After list-wise deletion of missing responses, our final sample size included in the path model was 3122.

Please see Table 1 for sample characteristics for all cancer survivor survey respondents, those that participated in LIVE**STRONG** physical activity events, and those that did not participate in LIVE**STRONG** physical activity events. A greater percentage of cancer survivors reporting physically active LIVE**STRONG** participation were men (52.6%) and identified as White (93.4%), as compared with those not participating in LIVE**STRONG** physical activity events (36.3, 89.0% respectively). Cancer survivors not engaged in LIVE**STRONG** physical activity events were more likely to self-identify as Black or African American as compared to cancer survivors reporting physically active LIVE**STRONG** participation, although only 1.7% of the entire sample self-identified as Black or African American (*n* = 53). Physically active LIVE**STRONG** participants were also more likely to report a longer survivorship length (*p* < .01) and a higher income (*p* < .001) than cancer survivors not participating in LIVE**STRONG** physical activity events. Cancer survivors engaged in physically active LIVE**STRONG** participation were also more likely to report a desire to help others, regular physical activity participation, and a higher quality of life, when compared with cancer survivors not participating in LIVE**STRONG** physical activity events (*p* < .001). No other sample characteristics were significantly different between cancer survivors who participated in LIVE**STRONG** physical activity events and those who did not.

**Table 1:** Descriptive characteristics of cancer survivors who participated in the LIVE**STRONG** 2010 Survey

	Overall Sample (*N* = 3122)	Physically active LIVESTRONG participant (*n* = 363)	Did not participate in LIVESTRONG in a physical active way (*n* = 2759)	Tests of difference between LIVESTRONG physical activity participants and others
Age	M = 48.2, SD = 12.7 (18–94)	M = 48.2, SD = 12.1 (18–80)	M = 48.2, SD = 12.7 (18–94)	*t* = .01, *p* > .05
Sex	*N* = 3122	*n* = 363	*n* = 2759	*t* = −5.88, *p* < .001
Female	1930 (61.8%)	172 (47.4%)	1758 (63.7%)	
Male	1192 (38.2%)	191 (52.6%)	1001 (36.3%)	
Survivorship Length	*N* = 3122	*n* = 363	*n* = 2759	χ^2^ = 11.63, *p* < .01
Finished treatment < 1 year ago	727 (23.3%)	64 (17.6%)	663 (24%)	
Finished treatment 1–5 years ago	1280 (41.0%)	143 (39.4%)	1137 (41.2%)	
Finished treatment ≥ 5 years ago	1115 (35.7%)	156 (43.0%)	959 (34.8%)	
Education	*N* = 3089	*n* = 360	*n* = 2749	*t* = .96, *p* > .05
H.S. Degree or less	270 (8.8%)	17 (4.7%)	253 (9%)	
Education > H.S. Degree	2819 (91.2%)	346 (95.3%)	2496 (91%)	
Income	*N* = 3122	*n* = 363	*n* = 2759	χ^2^ = 60.47, *p* < .001
$0 - $40,000	461 (14.8%)	29 (8.0%)	432 (15.7%)	
$41,000 - $60,000	405 (13.0%)	37 (10.2%)	368 (13.3%)	
$61,000 - $80,000	381 (12.2%)	43 (11.8%)	338 (12.3%)	
$81,000 - $100,000	376 (12.0%)	45 (12.4%)	331 (12%)	
$101,000 - $120,000	282 (9.0%)	24 (6.6%)	258 (9.4%)	
> $120,000	584 (18.7%)	118 (32.5%)	466 (16.9%)	
Preferred not to answer	633 (20.3%)	67 (18.5%)	566 (20.5%)	
Marital Status	*N* = 3092	*n* = 360	*n* = 2732	*t* = −.16, *p* > .05
Married / Domestic Partner	2123 (68.7%)	267 (73.6%)	1856 (68%)	
Other	969 (31.3%)	96 (26.4%)	876 (32%)	
Parental Status	*N* = 3116	*n* = 363	*n* = 2753	*t* = −.88, *p* > .05
No Children	1134 (36.4%)	122 (33.6%)	1012 (37%)	
≥ 1 Child	1982 (63.6%)	241 (66.4%)	1741 (63%)	
Race / Ethnicity^a^	*N* = 3122	*n* = 363	*n* = 2759	
American Indian or Alaskan Native	41 (1.3%)	2 (0.6%)	39 (1%)	*t* = −1.92, *p* > .05
Asian	57 (1.8%)	10 (2.8%)	47 (2%)	*t* = 1.18, *p* > .05
Black or African American	53 (1.7%)	1 (0.3%)	52 (2%)	*t* = −4.26, *p* < .001
Hispanic or Latino	146 (4.7%)	13 (3.6%)	133 (5%)	*t* = −1.17, *p* > .05
Native Hawaiian	7 (0.2%)	1 (0.3%)	6 (0.2%)	*t* = .22, *p* > .05
White	2801 (89.7%)	339 (93.4%)	2462 (89%)	*t* = 2.90, *p* < .01
Other	57 (1.8%)	5 (1.4%)	52 (1.9%)	*t* = −.80, *p* < .05
Refuse	46 (1.5%)	6 (1.7%)	40 (1.4%)	*t* = .30, *p* > .05
Desire to Help	*N* = 3122	*n* = 363	*n* = 2759	*t* = 3.18, *p* < .001
No	1514 (48.5%)	148 (40.8%)	1366 (49.5%)	
Yes	1608 (51.5%)	215 (59.2%)	1393 (50.5%)	
Regular Physical Activity Participation	*N* = 3122	*n* = 363	*n* = 2759	*t* = 9.48, *p* < .001
No	776 (24.9%)	36 (9.9%)	740 (26.8%)	
Yes	2346 (75.1%)	327 (90.1%)	2019 (73.2%)	
Quality of Life	*N* = 3122 M = 19.3, SD = 6.5	*n* = 363 M = 20.8, SD = 6.4	*n* = 2759 M = 19.1, SD = 6.5	*t* = 4.70, *p* < .001

N = sample size, M = mean, SD = standard deviation, H.S. = high school, QLACS = Quality of Life in Adult Cancer Survivors (31 indicates greater quality of life)

^a^Respondents could indicate multiple racial/ethnic groups; total % does not = 100%

Tests of difference between physically active LIVE**STRONG** participants and others: t-test for binary variables and χ^2−^test for multinomial variables

### Bivariate analyses

Bivariate analyses demonstrated that survivors’ physically active LIVE**STRONG** participation was positively correlated with having higher quality of life (*r* = .08, *p* < .01), participating in regular physical activity (*r* = .13, *p* < .01), and having a desire to help others through their cancer experience (*r* = .06, *p* < .01). Higher levels of physical activity engagement was also positively related to better quality of life (*r* = .18, *p* < .01). The relationships between the study variables were consistent with the expected directions. See Table 2.

**Table 2:** Bivariate correlations of study variables for cancer survivors participating in the 2010 LIVE**STRONG** participant survey

	Age	Sex	Survivorship	Income	Help	PAPL	PA	QOL
Age	1							
Sex^a^	.05^a^	1						
Survivorship^b^	.06^b^	.01	1					
Income^c^	.05^b^	−.05^b^	.02	1				
Help	−.23^c^	.00	−.07^c^	−.06^b^	1			
PAPL^d^	.00	−.11^c^	.06^b^	.08^c^	.06^b^	1		
PA^e^	−.00	−.08^c^	.05^b^	.06^c^	.05^b^	.13^c^	1	
QOL	.06^b^	−.27^c^	.10^c^	.17^c^	−.11^c^	.08^c^	.18^c^	1

*Note*. *n* = 3122. a dummy coded: 1 = male, 2 = female. b dummy coded: 0 = no, 1 = yes. b dummy coded: 1 = I finished treatment less than 1 year ago, 2 = I finished treatment between 1 and 5 years ago, 3 = I finished treatment 5 or more years ago, c: dummy coded: 0 = 0–40,000, 1 = 41,000–60,000, 2 = 61,000–80,000, 3 = 81,000–100,000, 4 = 101,000–120,000, 5 = 120,000 or more. d dummy coded: 0 = no, 1 = yes. e dummy coded: 0 = no, 1 = yes. ^a^*p* < .05. ^b^*p* < .01. ^c^*p* < .001. PAPL = Physically Active Participation in LIVE**STRONG**

*PA* = regular physical activity engagement. *QOL* = quality of life

### Model testing

**Fig. 2:**
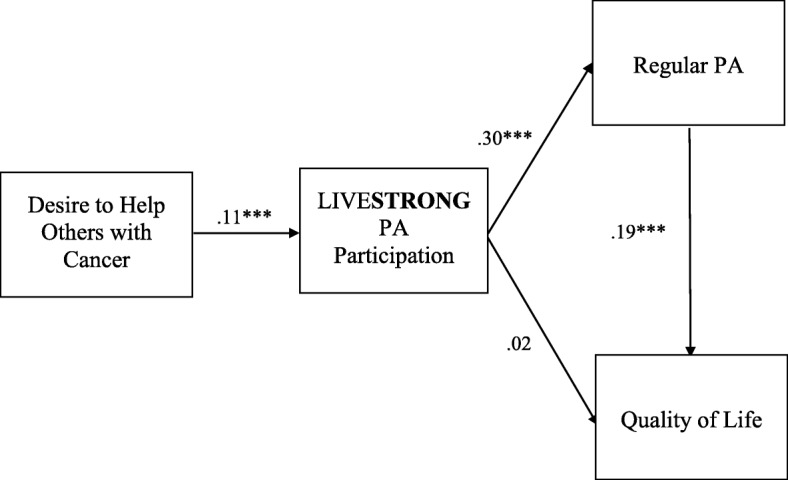
Path analysis of hypothesized relationships, controlling for age, sex, length of survivorship, and household income. Standardized path coefficients were reported. *n* = 3122; ***p < .001. PA = physical activity

To test the indirect effects of helping others on quality of life through physically active LIVE**STRONG** participation and regular physical activity, we added two additional direct paths to the model, bringing the model to be a just-identified (saturated) recursive model, and therefore yielding perfect fit indexes (see Fig. 2). Although such a model does not allow us to examine the goodness of fit, its advantage is that it is less likely to yield biased path coefficient estimates [62]. For this reason, just-identified models are appropriate when correctly estimating path coefficients is the primary concern [63], which fits with our study goal. Controlling for age, sex, income, and survivorship length, desire to help others was related with physically active LIVE**STRONG** participation (β = .11, *p* < .001), which was related with regular physical activity (β = .30, *p* < .001), and regular physical activity was related with higher quality of life (β = .194, *p* < .001).

In addition to these significant paths, the indirect effect of cancer survivors’ desire to help others on their quality of life via the two mediators (physically active LIVE**STRONG** participation and regular physical activity engagement) was significant (unstandardized indirect effect was.104 (se = .05), and 0 was not contained in the 95% bias-corrected confidence interval [.026, .233]). A closer look at the indirect effect also revealed that the specific indirect effect of desire to help others on quality of life was through first physically active LIVE**STRONG** participation and then regular physical activity engagement (.082 [.040, .105]), instead of through physically active LIVE**STRONG** participation only (−.053 [−.068, .126]). These results support the indirect effect of cancer survivors’ desire to help others on their quality of life through their physically active LIVE**STRONG** participation and then regular physical activity engagement.

To determine the robustness of the indirect effect that involved two sequential mediators (physically active LIVE**STRONG** participation and physical activity engagement), we conducted a sensitivity analysis using R [64] that helps determine when an observed indirect effect would disappear with the unobserved confounds included in the analysis [52]. We reported the R-squared value that indicates the proportions of the mediators and outcome variances explained by a hypothetical unobserved confound. A higher value of the R-squared indicates the importance of such a confound in the model. Our results showed that R-squared was .01, suggesting that the unobserved confound does not explain much of the indirect effect, and we therefore assert the robustness of the reported indirect effect.

## Discussion

Our results suggest that cancer survivors can personally benefit from participating in for-cause physical activity events, and that participating in this way could be a gateway to regular physical activity for these individuals, resulting positively on their quality of life. However, relatively few cancer survivors participate in for-cause physical activity events (11.4% in the present sample), and similar to non-cancer survivors participating in LIVE**STRONG** [65] and research examining physical activity engagement in the U.S. [66], more cancer survivors participating in for-cause physical activity events were men, self-identified as White, and reported higher income as compared to cancer survivors not participating in for-cause physical activity events. LIVE**STRONG** and many other cause-related organizations promote physical activity to help others, which links back to muscular Christian ideology, as early proponents advocated that the core ideology of muscular Christianity focuses on the transformation of society [37]. Original muscular Christian ideals of using one’s God-given body to help others was tied to the broader cultural push for social welfare in nineteenth century England. These ideals continue to persist within for-cause physically active philanthropy and health efforts today. Other popular examples of such physical activity related health efforts can be observed in the programs created by professional sport organizations in the United States, including *NFL Play 60*, *NBA Cares*, and the myriad of affiliates listed with MLBcommunity.org, including cancer and health related organizations. These professional sport organizations’ quality of life programs reflect underlying cultural desires and discourses around health and physical activity which are based on muscular Christian ideals. While we acknowledge there are many reasons cancer survivors may be motivated to participate in for-cause physical activity events, including regaining a sense of physical control or desire for community [67], we identified within this sample of cancer survivors that the desire to help others, a central tenant of muscular Christianity, was significantly related with this engagement, suggesting that this may be one reason cancer survivors are motivated to be physically active. As a social theology, muscular Christianity set forth the notion that sport and physical activity could make “good” out of the “bad” in society [37], and proponents of the movement believed that Christian moral values, learned through sport and physical activity, transferred to life beyond the playing fields [39, 68]. While making the “bad of society good” is a strong statement [34], this idea of doing good by helping others through using one’s physical body in physical activity or sport aligns with the rising movement of for-cause physical activity events, recently termed “physical philanthropy” [65].

Evidence supports the health benefits of physical activity and helping others for people who have been diagnosed with cancer and other diseases. There seems to exist important and overlapping personal benefits that have been experienced through both of these behaviors, including fewer or improved depressive symptoms, positive life engagement/purpose, lower anxiety, positive mental health, improvements in various physical health indicators, and greater quality of life [2–5, 20, 27, 29, 69, 70]. The present study examined how combining these two behaviors impacts both regular physical activity behavior and quality of life and concludes by suggesting that health-enhancing benefits can be gained through combining these efforts.

The LIVE**STRONG** Foundation and other cancer organizations should continue to encourage cancer survivors to help others through *physical philanthropy*, being physically active in these events. These organizations should also work to increase the number of cancer survivors participating in these events given the associated positive benefits of this type of participation for survivors themselves, specifically tailoring opportunities for those reporting less engagement (e.g., cancer survivors reporting a shorter survivorship length, women, Black or African Americans, and those reporting a lower income). Organizations that implement for-cause physical activity events should consider offering shorter distance events that might be more appealing and accessible to more people, including all cancer survivors and those less engaged as listed above. These shorter distance events would be more inclusive for participants of all physical health and those less comfortable/familiar with being active, while also providing “stepping stones,” as supported in hierarchical goal setting approaches, for cancer survivors and others to use as they strive to achieve goals of completing incrementally longer distance events in the future [71]. This would be especially beneficial for cancer survivors since reports suggest most are not meeting physical activity guidelines, reemphasizing the need for short-term, long-term, and incremental goals to increase physical activity engagement [72].

Researchers have emphasized that the complex nature of behavior change and sustaining positive behavior change, including physical activity, often requires a multi-faceted ecological approach [73, 74]. Aspects of for-cause physical activity events may be viewed through a social ecological lens and provide a translatable model for researchers and practitioners to use when promoting behavior change in a community. For example, for-cause physical activity events may have the potential to influence individual-level factors related with physical activity, such as self-efficacy, goal setting, intention, enjoyment, and motivation towards physical activity [53, 75, 76]. Due to the large numbers of participants in these events, individuals will also benefit through social support and forming relationships with members and leaders in the community [53, 77, 78]. As participation and interest in these organizational events continues to grow, guidelines and regulations of city centers and towns could potentially be adapted to support a physically active community (e.g., policies mandating investments in physical activity resources, for hosting physical activity events, open streets policies, or planning regulations to improve active transportation and/or leisure physical activity infrastructure [53, 79]).

### Limitations

Despite the encouraging findings suggested from the present study, several limitations need to be acknowledged. Given the cross-sectional design of data collection used for this analysis, causation could not be examined; and it is possible that more physically active cancer survivors were more likely to participate in physically active ways. Future research should use study designs that allow for temporality and causation of the variables of interest to be examined. Variables of interest were also measured using self-report, which includes inherent bias. Future research should also consider including objective measures of physical activity and more robust measures of helping others from other areas of research such as volunteerism and muscular Christianity to better understand the role of helping others when it comes to cancer survivors’ engagement in for-cause physical activity events, overall physical activity behaviors, and quality of life. Future work should aim to measure helping others with a multi-faceted scale, versus a single-item. And while informative, using a single-item physical activity measure was also not ideal; future research should employ more robust self-report measures capturing intensity, frequency, and duration where objective measures are not possible. It should also be noted that the data analyzed in this study was collected in 2010. While research continues to grow in the area of cancer survivorship and physical activity, the variables and pathways examined in the present study remain relevant today. Future work should confirm this in current data and further explore these relationships for people in various lengths of survivorship, those diagnosed with different types of cancers, and those seeking or who have experienced various treatment options. Additionally, these findings should be limited to the current sample given the low number of minority participants and the relatively young age of the sample (compared to overall survivorship age). Future researchers should intentionally recruit minority participants and older survivors to ensure representativeness. While the focus of the present study was to examine the presented relationships among cancer survivors, it is also possible that these same relationships exist for people never diagnosed with cancer. Thus, future research should examine the proposed relationships in a general adult population to further examine and understand these proposed relationships within our culture and how these relationships might be similar or dissimilar in these two groups. Physical activity history and physical activity levels prior to enrollment in the for-cause physical activity event should also be measured and considered as potential moderators in future work examining relationships presented in this study.

## Conclusions

Since this is the first study we are aware of that examines the proposed pathways between a desire to help others and positive health outcomes through using one’s physical body to do so, further research is needed to better understand these mediating relationships, as well as how to best address these relationships through intervention strategies. As discussed above, future research needs to include more detailed and robust measures of these constructs. It is also possible that qualitative approaches could help us better describe why these pathways are relevant to cancer survivors participating in physical philanthropy events. Researchers should consider conducting interviews and/or focus groups with physical philanthropy participants to examine the “whys” of their engagement. Finally, given that muscular Christian ideals are apparent in many facets of our culture today, and are posited to influence people’s everyday lives, it is plausible that the desire to help others is a determinant of many types of physical activity engagement, not just for-cause physical activity engagement. Future research should explore this possibility among cancer survivors and other populations, potentially comparing the relationships between desire to help others and physical activity among for-cause physical activity participants and other physical activity intervention participants. If there is a culturally solidified desire to help others through using our physical bodies, as suggested by muscular Christian ideology, then efforts toward understanding how to best harness these ideals for all physically philanthropic activities and incorporate them into future physical activity interventions should be explored. Finally, while our study suggests that helping others may be one motivator for cancer survivors to engage in physical activity, further research is needed to identify and better understand additional reasons why all cancer survivors, and specific subgroups of cancer survivors reporting less engagement (e.g., women, recent survivors, Black or African Americans, etc.), are or are not motivated to be physically active, and subsequently how to incorporate these factors into interventions and/or tailor intervention approaches.

To conclude, our results suggest that cancer survivors benefit from participating in for-cause physical activity events hosted by cancer organizations, including more regular physical activity and higher quality of life. Researchers need to further investigate the role of *helping others*, and cancer organizations should continue to encourage cancer survivors to *help others* in a multitude of ways including participation in for-cause physical activity events.

## Abbreviations

PA: Physical activity
